# Cardio-Oncology and Multi-Imaging Modalities

**DOI:** 10.3390/jcm14124353

**Published:** 2025-06-18

**Authors:** Christine M. Park, Ben Lerman, Felipe Contreras Yametti, Mario Garcia, Leandro Slipczuk, Aldo L. Schenone, Lili Zhang, Carlos A. Gongora

**Affiliations:** 1 Division of Cardiology, Montefiore Medical Center, Albert Einstein College of Medicine, Bronx, NY 10461, USA; chrpark@montefiore.org (C.M.P.); fecontrera@montefiore.org (F.C.Y.); mariogar@montefiore.org (M.G.); lslipczukb@montefiore.org (L.S.); lilizhan@montefiore.org (L.Z.); 2 Department of Medicine, Montefiore Medical Center, Albert Einstein College of Medicine, Bronx, NY 10461, USA; belerman@montefiore.org

**Keywords:** cardio-oncology, multi-modality imaging

## Abstract

Early detection and the rise of targeted cancer treatment have led to increased overall survival and decreased mortality among cancer patients. As the cancer survivor population ages, there is an increased risk for cardiovascular disease due to pre-existing comorbidities, deconditioning during therapy, or the natural progression of aging. Furthermore, with emerging oncologic therapies, there is an increased recognition of their potential cardiovascular toxicities. Indeed, heart disease is the leading cause of death in cancer survivors, which may reflect upon both the success of novel oncologic therapies and their potential cardiovascular toxicities. This recognition has driven the development of cardio-oncology, a multi-disciplinary field that involves collaboration between hematologists, oncologists, and cardiologists to screen, prevent, and manage cardiovascular disease in cancer patients and cancer survivors. The field focuses on early cardiovascular detection and prevention for these patients before, during, and after their oncologic treatment. As oncologic therapies evolve and our knowledge of short- and long-term adverse cardiovascular effects grows, it is critical for physicians to identify those at risk for increased morbidity and mortality, while also balancing the importance of their oncologic treatment plan. Multimodality cardiac imaging is the crux of the diagnosis and surveillance of these patients within cardio-oncology, and includes echocardiography, nuclear, computed tomography (CT), and cardiac magnetic resonance (CMR). Cardiac imaging is essential to establish the baseline function and assess various cardiotoxicities, including left ventricular dysfunction, heart failure, atherosclerosis, vascular injury, and arrhythmias. This review will discuss common oncologic therapies and their cardiotoxic profiles, the cardiac multimodality imaging modalities used in cardio-oncology, and the various approaches for the diagnosis and surveillance of this population.

## 1. Introduction

Early detection and the rise of targeted cancer treatment have led to increased overall survival and decreased mortality among cancer patients. As the cancer survivor population ages, they are at an increased risk for cardiovascular disease due to pre-existing comorbidities, deconditioning during therapy, and the natural progression of aging [1]. Furthermore, with emerging oncologic therapies, there is an increased recognition of their potential cardiovascular toxicities. Indeed, heart disease is the leading cause of death in cancer survivors, which may reflect upon both the success of novel oncologic therapies and their potential cardiovascular toxicities [2]. 

This recognition has driven the development of cardio-oncology, a multi-disciplinary field that involves collaboration between hematologists, oncologists, and cardiologists to screen, prevent, and manage cardiovascular disease in cancer patients and cancer survivors [3]. The field focuses on early cardiovascular detection and prevention for these patients before, during, and after their oncologic treatment. As oncologic therapies evolve and our knowledge of short- and long-term adverse cardiovascular effects grows, it is critical for physicians to identify those at risk for increased morbidity and mortality, while also balancing the importance of their oncologic treatment plan. 

Multimodality cardiac imaging is the crux of the diagnosis and surveillance of these patients within cardio-oncology, and includes echocardiography (ECHO), nuclear, computed tomography (CT), and cardiac magnetic resonance (CMR). Cardiac imaging is essential to establish a baseline function and assess various cardiotoxicities, including left ventricular dysfunction, heart failure, atherosclerosis, vascular injury, and arrhythmias [4]. This review will discuss common oncologic therapies and their cardiotoxic profiles, the cardiac multimodality imaging modalities used in cardio-oncology, and the various approaches for the diagnosis and surveillance of this population.

## 2. Cardiotoxicity and Cardiovascular Implications

The treatment options for cancer have increased substantially in the past several decades—novel therapies directly target the signaling pathways responsible for abnormal tumor cell proliferation, enhance the host’s immune system to target tumor antigens, or are directly cytotoxic by disrupting replication. However, the risk of the off-target effects remains and oncologic therapies can be associated with serious cardiovascular adverse events. Cardiotoxicities can range from systolic dysfunction, conduction abnormalities, or vascular and atherosclerotic complications. Below, we describe the common cardiotoxicities and their proposed mechanism of action from various cancer therapeutics and summarize them in Table 1. 

### 2.1. Cancer Therapy-Related Cardiac Dysfunction and Heart Failure

Cancer therapy-related cardiac dysfunction (CTRCD) is the most common and significant cardiotoxicity and is associated with many cancer therapies. It is broadly defined as a reduction in the cardiac function that can be asymptomatic or present as symptomatic heart failure. 

CTRCD has been linked to many cancer therapeutics; however, the two most commonly associated therapeutics causing CTRCD are anthracyclines and human epidermal growth factor receptor 2 (HER2) inhibitors. Anthracycline toxicity is generally dose-dependent and often presents within the first year of therapy initiation, with presentations ranging from asymptomatic dysfunction to clinical heart failure [5]. Conversely, HER2-inhibitor-related cardiac dysfunction is dose-independent and commonly manifests as reversible cardiomyopathy [6]. Anthracycline toxicity involves mitochondrial injury and DNA damage, while HER2 inhibitor cardiotoxicity involves the disruption of the protective function of HER2 in cardiomyocytes [5,7].

Diagnosis is based on imaging findings of decreased systolic function and symptoms of clinical heart failure. More specific thresholds for CTRCD have varied according to different guidelines and committees. In 2022, the American College of Cardiology Cardio-Oncology and Imaging Councils define *definite* CTRCD as a reduction in the left ventricular ejection fraction (LVEF) by ≥10% to a value <50% [2]. *Possible* CTRCD is defined as a reduction in the LVEF by ≥10% to a value of 50–55% or a reduction in the LVEF by <10% to a value of <50%, or a relative reduction in the ECHO global longitudinal strain (GLS) of ≥15% without change in the LVEF [2]. 

The management of CTRCD involves the addition of cardioprotective medications, such as beta-blockers and/or angiotensin-converting enzyme inhibitors. For patients with symptomatic or clinical heart failure from CTRCD, guideline-directed medical therapy is recommended. Once CTRCD is established, multidisciplinary discussions between the cardiologist and oncologist are crucial for cancer therapy planning. Understandably, the de-escalation or interruption of therapy can be stressful for patients, and care is taken to attempt to re-initiate therapy if deemed safe to do so. Thus, it is crucial that there is close communication within the cardio-oncology team when discussing interruptions to therapy or the re-initiation of therapy [8].

### 2.2. Myocarditis

Myocarditis has become an increasingly recognized cardiotoxicity due to the rise of immune checkpoint inhibitor (ICI) therapy. The incidence of ICI myocarditis is relatively low, with reports ranging from 0.1–1% [9]. However, the diagnosis can be challenging due to a wide range of clinical presentations. ICI myocarditis can range from mild, asymptomatic cases to fulminant, life-threatening myocarditis [9,10]. Since ICIs harness the host’s immune system to target tumor cells, adverse effects and toxicities are likely immune-mediated and caused by the activation of T cells that damage host tissues. ICI myocarditis is hypothesized to be due to T cell receptors targeting an antigen in the cardiac muscle that is similar to the tumor antigen. 

Diagnosis is based on symptoms (chest pain or shortness of breath), laboratory findings (elevated serum troponin and brain natriuretic peptide levels), electrocardiography findings, and/or cardiac imaging (such as CMR). The diagnosis of ICI myocarditis requires high clinical suspicion, as some mild cases of ICI myocarditis may only be revealed by abnormal laboratory markers or subtle electrocardiogram changes. Mortality from ICI can be as high as 38–46% in severe cases [11]. Treatment involves the cessation of the ICI therapy, supportive care and management, and in cases of refractory myocarditis, the initiation of high-dose steroids and even second-line immunosuppressants [12,13].

### 2.3. Conduction Disturbances 

Conduction disturbances have been associated with many oncological therapies, including radiation therapy, alkylating agents (such as cyclophosphamide and melphalan), and newer therapies such as the Chimeric antigen receptor T cell (CAR-T) and ICI [14]. Rhythm abnormalities that can develop include new-onset atrial fibrillation, bradyarrhythmias, and electrocardiogram changes such as QT prolongation. 

Specific mechanisms are not well-defined, but they may involve the development of inflammation and myocardial ischemia that interfere with cardiac conduction. Alternatively, conduction changes may be a sequelae of other cardiotoxicities, such as ICI myocarditis or vascular toxicities. Treatment depends on the type of conduction disturbance present. Atrial fibrillation is treated with the usual rate or rhythm control strategies, with anticoagulation given if indicated. For bradycardia and QT prolongation, treatment is less well-defined and involves the correction of electrolyte or metabolic disturbances and possible therapy discontinuation [15].

### 2.4. Autonomic Dysfunction

While related to rhythm abnormalities, autonomic dysfunction from oncological therapy is a distinct potential toxicity. Autonomic dysfunction commonly results from anthracyclines, platinum-based agents, taxanes, alkaloids, and radiation therapy [15]. Specific presentations vary and include orthostatic hypotension, chronotropic incompetence, and inappropriate sinus tachycardia. 

Proposed mechanisms include direct nerve damage from radiation and downstream inflammatory effects leading to nerve damage, electrolyte abnormalities, and cytokine release. Diagnosis involves autonomic function tests as well as laboratory markers, ECHO, and electrocardiograms. Treatment is dependent on the symptoms and includes lifestyle modification, midodrine or pyridostigmine, rate control with metoprolol or ivabradine (for inappropriate sinus tachycardia), or fullerenol or dexrazoxane, which may prevent anthracycline-associated cardiotoxicity [15].

### 2.5. Hypertension

Hypertension is a common comorbidity amongst cancer patients. Various oncological treatments, including anthracyclines and vascular endothelial growth factor (VEGF) inhibitors, often either exacerbate pre-existing hypertension or lead to a new diagnosis of hypertension. VEGF inhibitors have been shown to cause hypertension in a dose-dependent manner. The toxicity is inherent to VEGF inhibitors’ direct anticancer mechanism by increasing endothelin-1 and peripheral vascular resistance [16]. 

Diagnosis and surveillance often employ frequent blood pressure measurements, as well as routine cardiovascular monitoring, depending on the individual’s cardiac risk. In patients with hypertension associated with oncologic therapy, the first-line treatment is usually angiotensin-converting enzyme inhibitors, angiotensin receptor blockers, or calcium channel blockers.

### 2.6. Vascular Toxicity

Direct vascular toxicity can also be seen in VEGF inhibitors. This mainly manifests as a vasospasm, atherosclerosis, or arterial thrombotic events—such as thromboembolism—tissue infarction, or stroke. These vascular toxicities can also be exacerbated by the underlying hypercoagulable state of cancer patients [17]. Traditional therapies such as 5-flurouracil have been linked to vasospasms even in the absence of known coronary artery disease (CAD) [18]. Additionally, radiation therapy increases the risk for direct vascular toxicity, and can present many years after the completion of therapy [19]. Immunomodulator use (particularly thalidomide and lenalidomide in multiple myeloma) has a propensity for a higher rate of thrombotic events [17].

Diagnosis and treatment are usually imaging-based and directed at the underlying toxicity. A coronary vasospasm is managed with the usual vasodilatory agents such as nitrates or calcium channel blockers. Risk factor modification, such as CAD prevention with statin therapy, is also recommended for both primary and secondary prevention [20].

## 3. Cardiac Imaging Techniques in Cardio-Oncology

Cardiotoxicity from oncologic therapy requires prompt diagnosis, monitoring, and treatment [2]. It is important to assess the pre-treatment cardiac function, both to establish a baseline and assess the future risk. Imaging remains the crux of the diagnosis and surveillance of cardiotoxicities. Amongst the cardiac imaging modalities, transthoracic echo (TTE) is considered first-line and is performed at the baseline and during cancer treatment [4]. Other modalities include CMR, cardiac/coronary computed tomography (CCTA), and multi-gated acquisition (MUGA). The different cardiac imaging modalities have their individual strengths and roles in cardio-oncology, and the field continues to expand to make exciting advances in the detection, diagnosis, and management of cardiotoxicities. The strengths and limitations of each modality are outlined in Table 2. 

### 3.1. Echocardiography 

Echocardiography (ECHO) remains the first-line diagnostic imaging modality in cardio-oncology. Its widespread utility is owed to not only its ease in attainment, but also the breadth of information provided in terms of the function assessment, chamber sizing, valvular assessment, hemodynamic profiles, and evaluation of pericardial disease. Both TTE and transesophageal echocardiography (TEE) play a major role in detecting and managing cardiotoxicities. The most well-known and common cardiotoxicity is CTRCD, and ECHO provides a timely and accurate assessment of both the LVEF and right ventricular ejection fraction (RVEF). 

Two-dimensional (2D) ECHO was initially used to monitor the LVEF at the baseline and in interval periods during treatment. However, actual LVEF decline is now known as a later marker of CTRCD, and current protocols are utilizing ECHO strain techniques, diastolic assessment, and the integration of a three-dimensional (3D) volumetric assessment for the earlier detection and more accurate assessment of CTRCD [21].

Speckle-tracking ECHO, specifically “strain” techniques to assess left ventricular (LV) deformation, can provide quantitative information on the cardiac contractile function. GLS is an earlier and more sensitive marker of LV dysfunction than the decline seen in the LVEF and can detect subclinical LV dysfunction [22]. This has important prognostic information for patients [21,23]. In a meta-analysis assessing the prognostic value of GLS for early detection for CTRCD, a GLS decline of 13.6% predicted the future decline of the LVEF, and thus, the 15% GLS decline was established in guidelines in order to improve the specificity [21,24]. In LVEF calculations, GLS is more easily reproducible. Normal GLS values are <−18%, borderline values are −16% to −18%, and abnormal values are >−16%, which also variate from different vendors [25].

In addition to strain techniques, the 3D LVEF has emerged as the preferred LVEF assessment over 2D methods. A single-center observational cohort study compared 2D and 3D LVEF measurements to CMR-derived LVEF in patients with suspected CTRCD. The study found that 3D LVEF measurements correlated better with CMR-derived measurements than the 2D LVEF (3D LVEF −1.6% ± 6.3 vs. 2D LVEF −2.8% ± 6.3, *p* = 0.016), and led to the misclassification of CRTCD for approximately 10% of the total cohort [26]. Thus, the 3D LVEF is preferred if the technology/expertise is available.

**Figure 1:**
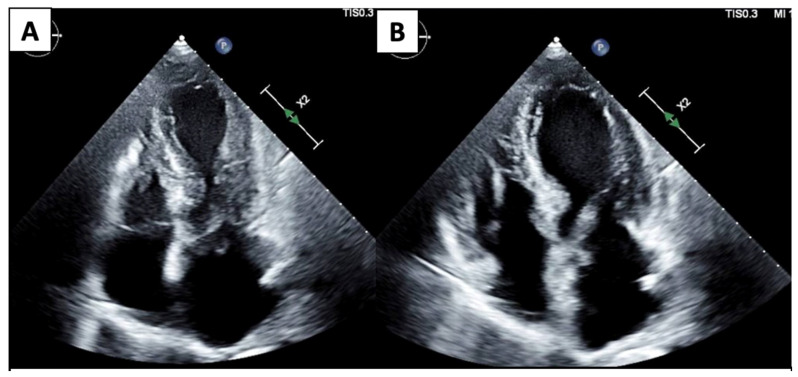
4 Chamber systole (**A**) and diastole (**B**) demonstrate apical dilation/ballooning and hypokinesis and basal contraction.

ECHO also has a wide range of use outside of monitoring for CTRCD. Given its ease of accessibility and comprehensive evaluation, it is often the first line imaging test for any cardiac-related presentation. TTE and TEE provide excellent imaging of valvular disease and allow for hemodynamic profiling using doppler methods. Cancer patients often suffer from pericardial diseases such as pericardial effusions, acute pericarditis due to cancer therapeutics or radiation therapy, or even direct tumor invasion into the pericardium [27]. ECHO is the first-line diagnostic tool for pericardial diseases and important hemodynamic information is provided using doppler ECHO to guide management and treatment decisions. Stress ECHO (exercise or pharmacologic) can be used in patients with an increased risk of atherosclerosis and vascular disease. Malignancies are often associated with stress cardiomyopathy, defined as an acute, transient decrease in the LV function associated with a stressor. ECHO is the imaging modality of choice to initially recognize stress cardiomyopathy, commonly referred to as “Takotsubo” cardiomyopathy (Figure 1), which can show the typical pattern of apical ballooning and akinesis/relative hypokinesis in comparison with the basal segments. Reverse stress cardiomyopathy patterns have also been described with basal hypokinesis and relative apical hyperkinesis [28]. 

### 3.2. Cardiac Magnetic Resonance

CMR imaging has emerged as a powerful, precise tool in cardio-oncology due to its established and well-proven reproducibility and accuracy in measuring cardiac chamber volumes, the functional assessment, and its various tissue characterization techniques. It is the standard reference for volumetric measurements and LV function assessments. However, CMR is limited by its availability and cost; the image quality can also be affected by devices/prostheses in the patient. 

CMR can be utilized if the patient has difficult echocardiographic windows or if there are discrepancies in the LV function assessment due to ECHO image acquisition or reader variability [29]. Additionally, CMR provides crucial tissue characterization techniques via T1/T2 mapping and late gadolinium enhancement evaluation (LGE), which have both diagnostic and prognostic implications for cardio-oncology patients. These CMR techniques can identify fibrosis and scar within the cardiac muscle, which has been shown to be early markers of myocardial injury in different cancer therapeutics [30]. CMR can also be applied to diagnose a spectrum of cardiac conditions, including ischemic heart disease via the scar pattern, infiltrative cardiomyopathies, and myocarditis via the Lake Louise Criteria [29].

**Figure 2:**
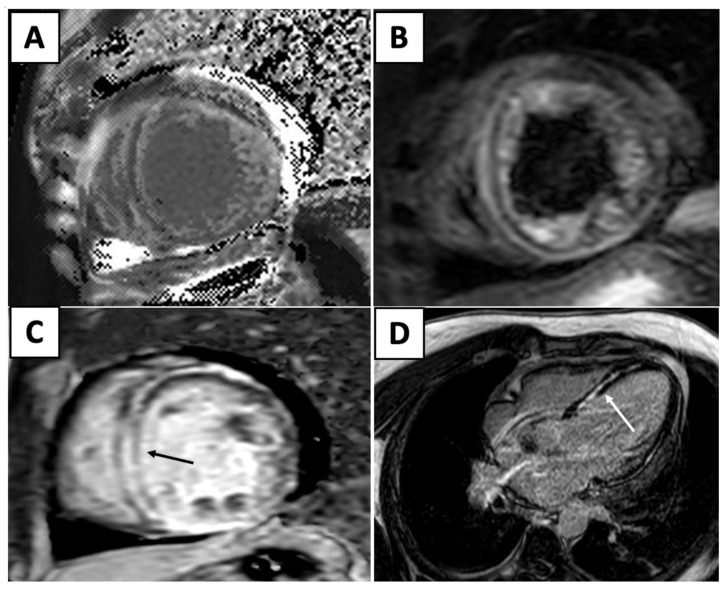
Elevated T1 mapping (**A**) and edema (**B**) in the T2 weighted images. (**C**) Short-axis (black arrow) and (**D**) four-chamber (white arrow) views reveal interventricular mid-myocardial LGE.

With the rise in immunotherapy in cancer therapeutics, there is increased recognition of immune checkpoint inhibitor (ICI) myocarditis. Due to the range of presentation, it can be particularly difficult recognizing ICI myocarditis. An endomyocardial biopsy is the gold standard of diagnosis for these patients, but owing to the invasive nature of this technique, CMR is a powerful, noninvasive technique that can accurately diagnose these patients. The original Lake Louise Criteria was established in 2009 and formalized the CMR diagnosis of myocarditis. However, since then, tissue characterization techniques have improved and many studies have demonstrated the incremental diagnostic utility of these techniques. Several studies demonstrated that T2 mapping techniques was useful to rule out acute myocardial inflammation, with a sensitivity reported as high as 89%. Although T2 mapping demonstrated better diagnostic accuracy in the acute myocarditis phase, other studies also showed that T1 mapping techniques are helpful for both acute and chronic myocarditis. T1 mapping techniques are also useful to rule out myocarditis, with a high negative predictive value of 92% [31]. Thus, the updated Lake Louise Criteria were published in 2018, and utilize multiparametric CMR techniques to combine the presence of inflammation/myocardial injury (T1-based marker), myocardial edema (T2-based), and the evaluation of LGE to diagnose myocarditis (Figure 2) [30].

**Figure 3:**
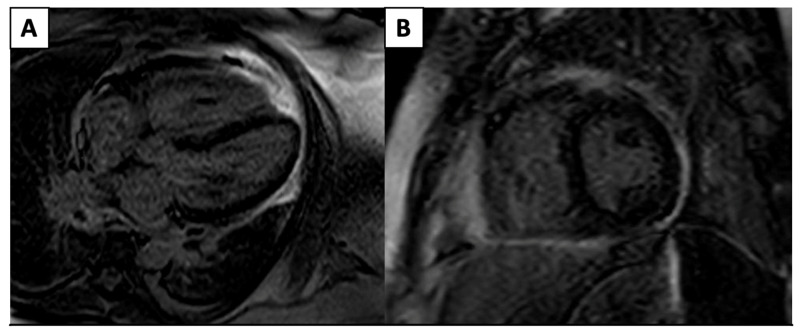
4 Chamber (**A**) and short-axis (**B**) phase-sensitive inversion recovery sequences that demonstrate severe circumferential pericardial LGE pericardial edema/inflammation.

Although ECHO remains first-line for pericardial diseases, CMR allows for a more accurate pericardial assessment via hemodynamics such as mitral and tricuspid inflow velocities and the direct visualization of pericardial thickening and LGE due to the presence of inflammation/edema (Figure 3), which has a moderately high sensitivity (65–100%) and high specificity (99–100%) across multiple studies [32]. 

Ongoing research and advances in CMR techniques are paving the way for a more cost-effective, timely, and widespread application of CMR within cardio-oncology. As such, it is the fastest-growing imaging modality within the field, with promising data on the earlier detection of cardiotoxicities and important prognostic implications.

### 3.3. Cardiac/Coronary Computed Tomography

Cardiac/coronary computed tomography angiography (CCTA) is commonly used as an effective and accurate non-invasive study for atherosclerotic heart disease, valvular disease, cardiac masses, and pericardial disease [33]. CCTA is relatively timely and cost-effective. 

**Figure 4:**
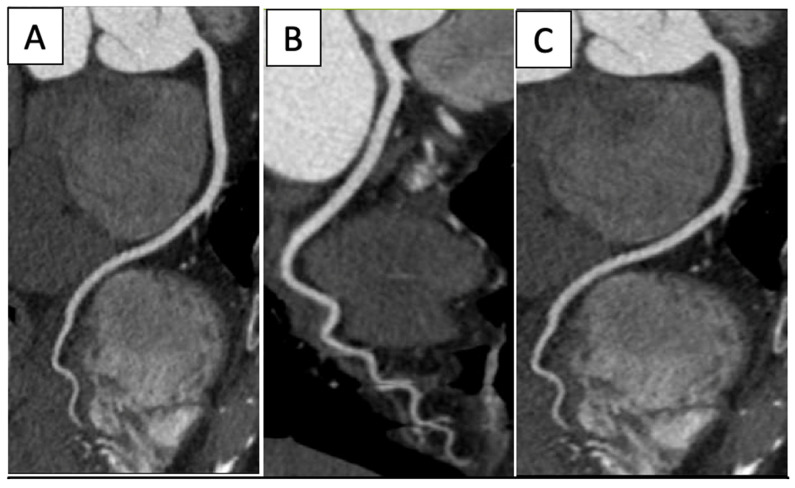
Normal coronary arteries’ CPR reconstructions: (**A**) Left anterior descendant, (**B**) Left circumflex, (**C**) Right coronary artery.

The effectiveness of CCTA for a coronary evaluation (Figure 4) and CAD has been well-established and studied [34] (Figure 4).

Non-contrast cardiac CT imaging can quantify coronary artery calcifications (CAC), which have been studied as risk predictors for future cardiac events. Therefore, cardiac CT is frequently utilized in the atherosclerotic disease risk stratification, screening, and evaluation of cardiac symptoms in oncology patients. Atherosclerotic disease is prevalent in oncology patients; certain cancer therapeutics have known vascular toxicities (i.e., radiation therapy) and cancer survivors are at a higher risk for atherosclerotic heart disease, which is the second leading cause of death among the survivors. In a substudy of the Multi-Ethnic Study of Athersoclerosis, the incidence of new CAC was independently associated with cancer history, with an increased relative risk of 1.3 among the large cohort [33,35]. Noninvasive coronary flow techniques, such as a CT fractional flow reserve (FFR), can be used to assess cardiac ischemia in order to minimize invasive strategies and guide further management on lesions that may be hemodynamically significant [33]. In a sub-analysis of a prospective cohort, the CT FFR had an improved area under the receiver–operative curve (0.94; 95% CI 0.92 to 0.96) when compared to CCTA alone (0.83; 95% CI 0.80–0.86; *p* < 0.001) or nuclear perfusion (0.70; 95% CI 0.65 to 0.74; *p* < 0.001) [36].

CCTA is also used for percutaneous and surgical planning for concomitant valvular disease in the cancer patient. For example, patients with carcinoid syndrome can have associated tricuspid valvular lesions that can be amendable to intervention. Prior history of radiation therapy is also associated with valve degeneration, and CCTA is routinely utilized for percutaneous and surgical planning. 

**Figure 5:**
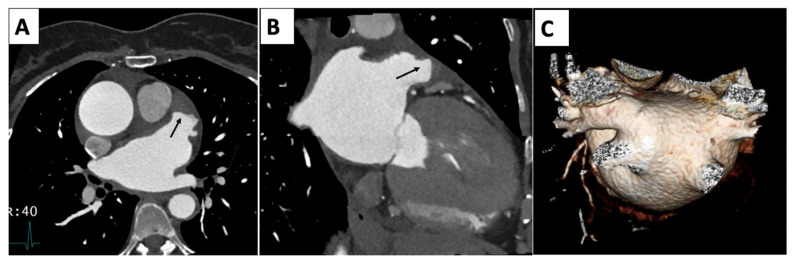
Multiplanar reconstruction (**A**,**B**) with arrows demonstrating a thrombus in the tip left-atrial appendage. (**C**) Volume rendering 3D reconstruction of left atrium with separate insertion of the right-superior and -inferior and left-superior and -inferior pulmonary arteries.

CCTA has also become an essential imaging tool in the diagnosis and evaluation of a cardiac thrombus, offering several advantages over traditional diagnostic methods like ECHO, which provides high-resolution images that can effectively detect a cardiac thrombus, especially in complex anatomical regions like the left atrium, left ventricle, or in cases of atrial fibrillation (Figure 5). 

CCTA offers a non-invasive, accurate, and detailed approach for diagnosing and evaluating a cardiac thrombus, providing essential information for treatment planning and risk management in patients [37]. 

Finally, CCTA can identify the presence of pericardial effusion, pericardial calcification, or the direct tumor invasion of the pericardium. CCTA allows for the full visualization and imaging of the entire pericardium and is relatively more rapid and cost-effective than CMR. 

### 3.4. Nuclear Imaging

Nuclear imaging techniques, such as single-photon-emission computed tomography (SPECT) and positron emission tomography (PET), have a long-established role in the assessment of myocardial perfusion (Table 3). Vascular toxicities of cancer therapeutics are well-known and nuclear imaging techniques can be considered in the functional assessment of these patients. MUGA scanning was used previously for ECHO in LVEF assessments, but now is only utilized when ECHO and CMR are not available [4].

PET is frequently utilized by the oncologist to assess the metastasis and tumor burden. It can lead to the diagnosis of cardiac metastasis or a primary cardiac malignancy. Transthyretin cardiac amyloidosis is diagnosed via a technetium pyrophosphate scan. Nuclear imaging is relatively widespread (with some limitation in the PET availability), but due to its high radiation exposure, its use is not as widespread as ECHO, CMR, or CT.

### 3.5. Cardiac Masses

Cardiac masses encompass a diverse spectrum of pathologies, encompassing both benign and malignant tumors. Cardiac masses are often asymptomatic; however, as they increase in size and invade the structures of the heart, they can cause significant hemodynamic effects. Thus, cardiac imaging plays a pivotal role in both early detection and characterization, which can inform the management of these tumors. Due to its widespread accessibility, ECHO is the most prevalent modality for the initial detection of cardiac masses, which frequently manifest as incidental findings [4]. ECHO can localize masses, identify salient characteristics such as the shape and size, and assess for hemodynamic effects of the mass. Although ECHO is usually first-line due to its ubiquity and ease in attainment, CMR remains the noninvasive gold standard in the diagnosis of cardiac masses. CMR allows for advanced tissue characterization techniques that are comparable to histology. Its superior resolution also facilitates the detection of infiltration into surrounding tissues. CT provides precise multiplanar anatomic detail, which is beneficial for pre-surgical planning [33], while PET aids in distinguishing between benign and malignant masses and provides staging information [38]. Consequently, the accurate diagnosis of cardiac masses necessitates a comprehensive multimodality imaging approach.

## 4. Imaging for Oncological Therapies at Baseline and Follow-Up

Cardiac imaging is the crux of surveillance for patients undergoing cancer therapeutics. Depending on the cancer therapy, there are different recommendations regarding the frequency of testing. ECHO remains first-line for all the therapies below. However, if the patient has poor ECHO windows, or if there are discrepancies in the assessment, CMR is recommended as the next-line imaging modality. In instances when CMR is unavailable, MUGA is used as a third-line diagnostic tool. The recommendations of imaging at the baseline and follow-up are highlighted by cancer therapeutic in Table 1. 

### 4.1. Anthracyclines

The most well-known category of cardiotoxic chemotherapy is anthracyclines. Anthracyclines, such as doxorubicin, epirubicin, and idarubicin, are used for a variety of cancers, including lymphoma, leukemia, and breast cancer. Their cardiotoxicity is dose-dependent and often manifests shortly after therapy initiation as CTRCD, or symptomatic heart failure with a decreased LV function [5]. Anthracyclines inhibit Topoisomerase-IIβ in cardiomyocytes, which leads to DNA double-strand breaks and cell death, free-radical creation, and lipid peroxidation, mitochondrial damage, and apoptosis in cardiomyocytes [5]. 

Anthracyclines have a well-established high risk for CTRCD, and patients undergoing treatment with one or multiple types of anthracyclines encompass a high proportion of cardio-oncology patients. ECHO is used for the baseline assessment and surveillance of these patients [21].

The recommendations for cardiotoxicity monitoring for patients undergoing therapy with anthracyclines include baseline ECHO and interval assessments during treatment. The baseline ECHO should ideally be performed 3 months prior to initiating cancer therapy and is a full, comprehensive study. The interval assessment can be more focused and the time interval can depend on the patient’s risk factors, the length of the cancer therapy treatment, and the result of the baseline study. The frequency of serial testing varies across guidelines and expert opinion. Generally, for patients on anthracyclines, due to their dose-dependent cardiotoxicity risk, a follow-up assessment is recommended after the cumulative dose reaches >250 mg/m^2^ doxorubicin-equivalent and again every 50 mg/m^2^ afterwards [21]. After treatment is completed, post-treatment surveillance is recommended for 1 to 5 years after completion. Post-treatment surveillance also depends on whether the patient developed CTRCD, the cancer-related prognosis, and presence of other cardiac and non-cardiac risk factors. 

The standard ECHO protocol for the baseline assessment prior to initiating cancer treatment with anthracyclines should include 2D and 3D protocols for ventricular volumes and the LVEF calculation, the right ventricle size and RVEF assessment, speckle-tracking ECHO with global longitudinal strain (GLS), and diastology [4,21]. In addition to the systolic function assessment, a diastolic evaluation is recommended, as diastolic dysfunction can often precede systolic dysfunction in CTRCD [39,40].

### 4.2. HER2-Targeted Monoclonal Antibodies

HER2 inhibitors have been used to treat HER2-positive breast cancer and stomach cancer [6,7]. Trastuzumab, a monoclonal antibody, is the most commonly used HER2 inhibitor. These drugs bind to the HER2 extracellular domain and inhibit the proliferation of cells that overexpress HER2. They disrupt protective HER2 signaling, which increases cardiomyocyte vulnerability to stress, decreases cardiomyocyte energy production, and reduces contractility [41]. Unlike irreversible structural damage caused by anthracyclines, HER2 inhibitor-related cardiotoxicity does not typically involve direct myocyte destruction. Their toxicity often presents as an asymptomatic decrease in the LVEF and can reverse after drug discontinuation [7].

Similar to the recommendations for anthracycline cardiotoxicity, patients on HER2-targeted monoclonal antibodies should undergo baseline ECHO and interval assessments during treatment. Unlike anthracycline cardiotoxicity, HER2 cardiotoxicity is not dose-dependent. Thus, during active HER2-targeted treatment, ECHO is recommended once every 3 months for surveillance. Due to the reversibility of the cardiotoxicity and rarity of late-presenting adverse effects, patients who have completed or discontinued HER2 therapy do not need routine TTE screening after the cessation of therapy.

### 4.3. Proteasome Inhibitors 

Proteasome inhibitors, such as bortezomib or carfilzomib, are associated with cardiotoxic effects. These therapeutics, commonly used in the treatment of multiple myeloma, can lead to the development of heart failure, ischemic heart disease, or dysrhythmias [42]. Cardiac imaging is recommended for a baseline assessment prior to initiating proteasome inhibitor therapy. ECHO is first-line, with LV systolic and diastolic function assessments, RVEF, and GLS [42]. For patients receiving carfilzomib, the European Society of Cardiology guidelines in 2022 recommend surveillance TTE every three cycles (a class 2a recommendation for high-risk patients and class 2b recommendation for low–moderate-risk patients) [8]. Otherwise, during treatment with other proteasome inhibitors (i.e., bortezomib), surveillance imaging is only indicated if the patient experiences any cardiac-related symptoms. 

### 4.4. Combination Therapy of BRAF and MEK Inhibitors

BRAF and MEK inhibitors are used in combination to treat non-small-cell lung cancer and melanoma, which harbor BRAF mutations. Common BRAF inhibitors include dabrafenib and encorafenib, while MEK inhibitors include trametinib and binimetinib. Combination therapy with BRAF and MEK inhibitors are more effect clinically in oncologic treatment; however, there is an increased risk of cardiotoxic effects [43]. These inhibitors can impair the mitochondrial integrity and increase oxidative stress, which leads to cell apoptosis, cellular dysfunction, and reduced myocardial contractility. 

The primary cardiotoxic effects of combination therapy with BRAF inhibitors and MEK inhibitors include a risk for the development of hypertension, CTRCD, pulmonary embolus, or QT prolongation (particularly when used in the setting of other known QT-prolonging drugs). Combination BRAF and MEK therapy has an increased risk for cardiotoxicity compared to monotherapy with BRAF alone [44]. Baseline ECHO is recommended in all patients who will receive combination BRAF and MEK inhibitor therapy. Surveillance imaging with ECHO is recommended 1 month after initiating therapy, then every 2–4 months while on therapy, depending on the patient’s risk profile [8,44].

### 4.5. Select VEGF Inhibitors

Certain VEGF inhibitors (i.e., pazopanib and sunitinib) are associated with heart failure, atherosclerotic heart disease, or the development of hypertension [42,45]. Since tumor growth depends on new blood vessel formation, VEGF inhibitors are widely utilized in various malignancies for tumor regression. However, it is not surprising that there are cardiac side effects, given the importance of vasculature in cardiac function. The loss of microvasculature can lead to an increase in blood pressure, which can develop relatively rapidly after the initiation of therapy [16]. Cardiac ischemia, arterial thrombosis, and the development of atherosclerosis have also been reported [16]. 

A baseline assessment with ECHO is recommended prior to the initiation of therapy. The reassessment of the LV systolic function is recommended after cycle 1 and can also be repeated after cycle 3. The new development of cardiotoxic effects is rare after cycle 3 if not already present. After cycle 3, interval imaging can be carried out only if symptoms arise in an otherwise asymptomatic patient. 

Afliberecpt, a VEGF inhibitor, is associated with heart failure, the development of hypertension, and an increased risk for an arterial thromboembolism. A baseline assessment with ECHO is recommended prior to the initiation of therapy, and then repeat imaging is only needed with the development of concerning symptoms [4,46].

### 4.6. Tyrosine Kinase Inhibitors

Target kinase inhibitors (TKI) target multiple signal transduction cascades, allowing for various cancer-inhibiting effects. Some examples include Osimertinib—which irreversibly binds the EGFR kinase and, therefore, blocks signaling pathways downstream— imatinib—which targets the BCR-ABL fusion protein in chronic myelogenous leukemia— and ibrutinib—which targets the Bruton’s tyrosine kinase, a key enzyme in the BCR pathway. 

Select TKI such as osimertinib (EGFR-TKI) and ibrutinib (Bruton TKI) have associated cardiotoxic effects. Osimertinib is associated with the development of heart failure, atrial fibrillation, or QT prolongation, whereas ibrutinib can lead to the development of atrial fibrillation, ventricular arrhythmias, or heart failure. For both TKI drugs, a baseline assessment with ECHO is recommended prior to therapy. For osimertinib, 3-month interval ECHO is recommended while undergoing treatment. For ibrutinib, repeat cardiac imaging is recommended only if symptoms arise [4].

### 4.7. Immune Checkpoint Inhibitors (ICI)

ICI are a novel, emerging therapy that have radically shifted the treatment of a range of solid and hematological tumors. ICI work by enhancing the body’s antitumor immune response. The three main subtypes are inhibitors of cytotoxic T-lymphocyte-associated antigen-4 (CTLA-4), programed cell death-1 (PD-1), and its ligand (PD-L1) [47]. PD-1 and PD-L1 inhibitors function by reinitiating an immune response that tumors may circumvent. CTLA-4 functions by inhibiting an immune response and their inhibitors can restore that response. ICI may elicit immune-related inflammatory adverse events, which includes myocarditis and pericarditis. 

ICI are associated with the development of arrhythmias and myocarditis, which can have a varied presentation from mild, asymptomatic cases to fulminant myocarditis [9]. A baseline assessment with ECHO is recommended prior to initiating therapy. If symptoms arise, ECHO is considered first-line; however, CMR has proven utility in the diagnosis of and has shown prognostication value for ICI-associated myocarditis [9].

### 4.8. CAR T Cell Therapy

CAR-T therapy is a new, emerging therapy for B cell lymphomas and multiple myeloma. CAR-T therapy modifies the patient’s T cells to express chimeric antigen receptors that can target specific cancer cells [48]. 

CAR-T therapy has been associated with a higher risk of major adverse cardiovascular events (MACE) and the development of heart failure [49]. In a retrospective study examining MACE in patients receiving CAR T cell therapy, the development of grade 3 or 4 cytokine-release syndrome (CRS), a systemic severe inflammatory response to the CAR-T infusion, was independently associated with a higher proportion of MACE. 

A baseline assessment with ECHO is recommended; the development of CRS grade 2 or higher warrants repeat the assessment with ECHO. It is also postulated that the release of cytokines in CRS can cause direct myocardial injury [49].

### 4.9. Alkylating Agents

Alkylating agents such as mitomycin, busulfan, cyclophosphamide, and melphalan are commonly used for hematological and oncological cancers [14]. They preferentially target rapidly dividing cells and attach an alkyl group that leads to cytotoxicity and cell death [50]. Alkylating agents can damage endothelial cells and trigger an inflammatory response within the myocardium, which can reduce oxygen delivery to cardiomyocytes and precipitate myocardial dysfunction. In addition, they generate free radicals and disrupt the mitochondrial function which damages the cardiomyocytes. They may lead to acute cardiotoxicity (such as acute heart failure or hemorrhagic myocarditis) or induce chronic cardiac dysfunction. Low-grade toxicity may improve upon therapy cessation, whereas severe acute damage is less likely to fully reverse. 

Mitomycin is associated with CTRCD in a dose-dependent manner. Busulfan and melphalan have been linked to arrhythmia and vascular toxicity, while cyclophosphamide is linked to direct cardiac toxicity, with reports of life-threatening hemorrhagic myocarditis at high doses [51,52].

For mitomycin, a baseline assessment with ECHO is recommended prior to the initiation of the therapy and repeat interval imaging should be carried out if there are symptoms which are concerning for CTRCD [53].

## 5. Future Directions

Cardio-oncology is rapidly advancing toward a more personalized, proactive, and preventive model of care, driven by innovations in diagnostics, therapeutics, and interdisciplinary collaboration. Future directions in the field emphasize the integration of multiple technologies to identify patients at the highest risk for CTRCD. The development of predictive biomarkers and advanced imaging techniques, such as strain ECHO and cardiac MRI with tissue characterization, is enabling the earlier detection of subclinical cardiac dysfunction. Artificial intelligence and machine learning are increasingly being applied to large datasets to enhance risk stratification, guide therapeutic decisions, and predict adverse outcomes. On the therapeutic front, novel cardioprotective agents are being investigated to reduce the risk of CTRCD. One of the most promising areas of innovation involves the use of sodium–glucose cotransporter-2 (SGLT2) inhibitors, originally developed for type 2 diabetes, but now recognized for their robust cardioprotective properties [54]. These agents are being investigated and our group has demonstrated their potential. Our prior research showed that SGL2 inhibitors are safe in cancer patients and reduce cardiac events in patients treated with anthracyclines [55]. Future clinical trials and translational studies will be crucial to define the optimal timing, patient selection, and integration of SGLT2 inhibitors within the broader cardio-oncology treatment landscape.

Also, survivorship programs and long-term cardio-oncology follow-up are gaining attention to address the growing population of cancer survivors at risk for late-onset cardiovascular disease. Lastly, the field of cardiogenetics is continuously growing and there are studies that are looking into relationships into genetics and CTRCD [56]. Together, these innovations aim to refine the balance between effective cancer treatment and cardiovascular health, ultimately improving both the survival and quality of life for oncology patients.

## 6. Conclusions

The increase in cancer survivorship is owed to the explosion of novel cancer therapeutics that has emerged in the last few decades. With these promising advances within oncology comes the increased recognition of the potential cardiotoxicities that can affect both current patients and cancer survivors. Cardio-oncology focuses on the multidisciplinary management of this special population, and the role of advanced cardiac imaging is crucial to the diagnosis, surveillance, and management of cardiotoxicities. As we continue to push the boundaries of oncologic therapeutics, it is also equally important to advance our knowledge of the potential cardiotoxicities that may occur and continue to integrate cardiac imaging to better detect and manage the cardiovascular risk in these patients. 

## Figures and Tables

**Table 1:** Cardiac imaging assessment recommendations based on cancer therapeutic. Adapted from Baldassarre, LA et al., “Advances in Multimodality Imaging in Cardio-Oncology”: JACC State-of-the-Art Review [4].

Therapeutic	Cardiotoxicity	Baseline and Follow-Up
Anthracyclines	Heart failure *, cardiomyopathy, arrhythmias	Baseline: TTE with 2D, 3D, GLS, diastolic function Follow-Up: Repeat TTE when cumulative dose >250 mg/m^2^ doxorubicin-equivalence and after every 50 mg/m^2^. Then, serial imaging from 1-5 years after therapy.
HER2-targeted monoclonal antibodies	Heart failure *, cardiomyopathy	Baseline: TTE with 2D, 3D, GLS, diastolic function Follow-Up: Repeat TTE every 3 months
Proteasome inhibitors	Heart failure *, ischemic heart disease, dysrhythmias	Baseline: TTE with 2D, 3D, GLS, diastolic function Follow-Up: Repeat if symptomatic. For carfilzomib, repeat TTE every 3 cycles
Combination therapy with BRAF + MEK inhibitors	Hypertension *, heart failure, QT prolongation, pulmonary embolus	Baseline: TTE with 2D, 3D, GLS, diastolic function Follow-Up: Repeat TTE one month after initiation of treatment, then every 2-4 months
VEGF inhibitors	Heart failure *, ischemic heart disease, hypertension	Baseline: TTE with 2D, 3D, GLS, diastolic function Follow-Up: Reassessment of LVEF after cycle 1 and cycle 3. Repeat if symptomatic
Tyrosine kinase inhibitors	Heart failure *, atrial fibrillation, ventricular arrhythmias, or QT prolongation	Baseline: TTE with 2D, 3D, GLS, diastolic function Follow-Up: 1) Osimertinib, repeat TTE every 3 months. 2) Ibrutinib, repeat only if symptomatic
Immune checkpoint inhibitors	Arrhythmias *, myocarditis, pericarditis	Baseline: TTE with 2D, 3D, GLS, diastolic function Follow-Up: Only if symptomatic
CAR T-cell therapy	Heart failure *, tachyarrhythmias, cardiomyopathy	Baseline: TTE with 2D, 3D, GLS, diastolic function Follow-Up: Repeat TTE if there is CRS grade 2 or higher, or if symptomatic

Trans-thoracic echocardiogram (TTE), global longitudinal strain (GLS), * = Designates major cardiotoxic effect.

**Table 2:** The main imaging modalities in cardio-oncology are ECHO, CMR, CT, and nuclear, with their strengths and limitations.

Imaging Modality	Sequence	Strengths	Limitations
Echocardiography (TTE, TEE)	2D, 3D volumetric assessment Diastolic function GLS Valvular assessment Hemodynamic profiles (i.e., pulmonary hypertension)	First-line imaging Cost-effective Complete evaluation of cardiac structures and function	Operator-dependent Certain patients can have technically difficult windows
Cardiac MRI	Cine sequences, LV mass and volume measurements Valvular assessment Multiparametric tissue characterization with T1, T2, ECV Strain techniques and stress LGE CMR available	Superior spatial and temporal resolution Complete evaluation of cardiac structures The standard for evaluation of cardiac function and volumes	Expensive, less accessible Images can be degraded by intra-body devices/prostheses Can be limited in patients with end-stage renal disease or claustrophobia
Cardiac CT	Contrast perfusion for coronary CTA FFR evaluation Non-contrast CT sequences for calcium (coronary calcium) evaluation	Cost-effective, timely, widespread Relatively small amounts of radiation	Use of iodinated contrast, requires gating with ECG, and can be limited in patients with high heart rate
Nuclear	MUGA Stress SPECT or PET, Technetium pyrophosphate scan	Widespread access Functional assessment of ischemic heart disease	High amount of radiation, limited evaluation of cardiac structures, presence of attenuation artifact, limited availability for PET

**Table 3:** Illustrations of imaging modalities in cardio-oncology ECHO, CMR, CT, and nuclear.

Imaging Modality	Imaging Illustrations	Description
Echocardiography	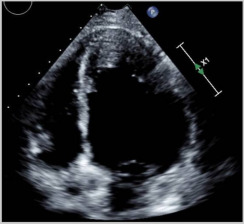	Mild left-ventricular dilation and systolic dysfunction
Cardiac MRI	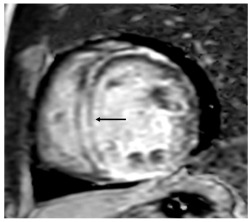	Short-axis view reveal interventricular mid-myocardial LGE (black arrow).
Cardiac CT	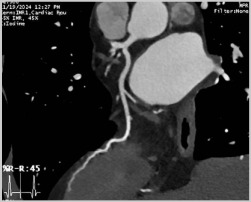	Left-anterior descending artery on cardiac CT
Nuclear	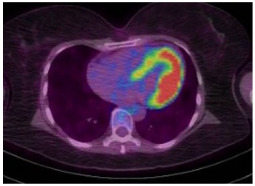	Normal fluorodeoxyglucose uptake in the myocardium

## Abbreviations

Computed tomography	(CT)
Cardiac magnetic resonance	(CMR)
Cancer treatment-related cardiac dysfunction	(CTRCD)
Human epidermal growth factor receptor 2	(HER2)
Left-ventricular ejection fraction	(LVEF)
Global longitudinal strain	(GLS)
Immune checkpoint inhibitor	(ICI)
Chimeric antigen receptor T cell	(CAR-T)
Vascular endothelial growth factor	(VEGF)
Coronary artery disease	(CAD)
Transthoracic echocardiography	(TTE)
Cardiac/coronary computed tomography	(CCTA)
Multi-gated acquisition	(MUGA)
Transesophageal echocardiography	(TEE)
Right-ventricular ejection fraction	(RVEF)
Left-ventricular	(LV)
Two-dimensional	(2D)
Three-dimensional	(3D)
Late gadolinium enhancement	(LGE)
Coronary computed tomography angiography	(CCTA)
Coronary artery calcification	(CAC)
Fractional flow reserve	(FFR)
Single-photon-emission computed tomography	(SPECT)
Positron emission tomography	(PET)
Target kinase inhibitors	(TKI)
Cytotoxic T-lymphocyte-associated antigen-4	(CTLA-4)
Programmed cell death-1	(PD-1)
Major adverse cardiovascular events	(MACE)
Cytokine release syndrome	(CRS)

## References

[1] Lenneman C.G., Sawyer D.B. (2016). Cardio-Oncology: An Update on Cardiotoxicity of Cancer-Related Treatment. Circ. Res..

[2] Herrmann J., Lenihan D., Armenian S., Barac A., Blaes A., Cardinale D., Carver J., Dent S., Ky B., Lyon A.R. (2022). Defining Cardiovascular Toxicities of Cancer Therapies: An International Cardio-Oncology Society (IC-OS) Consensus Statement. Eur. Heart J..

[3] Herrmann J., Lerman A., Sandhu N.P., Villarraga H.R., Mulvagh S.L., Kohli M. (2014). Evaluation and Management of Patients with Heart Disease and Cancer: Cardio-Oncology. Mayo Clin. Proc..

[4] Baldassarre L.A., Ganatra S., Lopez-Mattei J., Yang E.H., Zaha V.G., Wong T.C., Ayoub C., DeCara J.M., Dent S., Deswal A. (2022). Advances in Multimodality Imaging in Cardio-Oncology: JACC State-of-the-Art Review. J. Am. Coll. Cardiol..

[5] Camilli M., Cipolla C.M., Dent S., Minotti G., Cardinale D.M. (2024). Anthracycline Cardiotoxicity in Adult Cancer Patients: JACC: CardioOncology State-of-the-Art Review. JACC CardioOncol..

[6] Dempsey N., Rosenthal A., Dabas N., Kropotova Y., Lippman M., Bishopric N.H. (2021). Trastuzumab-Induced Cardiotoxicity: A Review of Clinical Risk Factors, Pharmacologic Prevention, and Cardiotoxicity of Other HER2-Directed Therapies. Breast Cancer Res. Treat..

[7] Wu Q., Bai B., Tian C., Li D., Yu H., Song B., Li B., Chu X. (2022). The Molecular Mechanisms of Cardiotoxicity Induced by HER2, VEGF, and Tyrosine Kinase Inhibitors: An Updated Review. Cardiovasc. Drugs Ther..

[8] Lyon A.R., Lopez-Fernandez T., Couch L.S., Asteggiano R., Aznar M.C., Bergler-Klein J., Boriani G., Cardinale D., Cordoba R., Cosyns B. (2022). 2022 ESC Guidelines on Cardio-Oncology Developed in Collaboration with the European Hematology Association (EHA), the European Society for Therapeutic Radiology and Oncology (ESTRO) and the International Cardio-Oncology Society (IC-OS). Eur. Heart J..

[9] Mahmood S.S., Fradley M.G., Cohen J.V., Nohria A., Reynolds K.L., Heinzerling L.M., Sullivan R.J., Damrongwatanasuk R., Chen C.L., Gupta D. (2018). Myocarditis in Patients Treated with Immune Checkpoint Inhibitors. J. Am. Coll. Cardiol..

[10] Zhang L., Awadalla M., Mahmood S.S., Nohria A., Hassan M.Z.O., Thuny F., Zlotoff D.A., Murphy S.P., Stone J.R., Golden D.L.A. (2020). Cardiovascular Magnetic Resonance in Immune Checkpoint Inhibitor-Associated Myocarditis. Eur. Heart J..

[11] Frascaro F., Bianchi N., Sanguettoli F., Marchini F., Meossi S., Zanarelli L., Tonet E., Serenelli M., Guardigli G., Campo G. (2023). Immune Checkpoint Inhibitors-Associated Myocarditis: Diagnosis, Treatment and Current Status on Rechallenge. J. Clin. Med..

[12] Brahmer J.R., Abu-Sbeih H., Ascierto P.A., Brufsky J., Cappelli L.C., Cortazar F.B., Gerber D.E., Hamad L., Hansen E., Johnson D.B. (2021). Society for Immunotherapy of Cancer (SITC) Clinical Practice Guideline on Immune Checkpoint Inhibitor-Related Adverse Events. J. Immunother. Cancer.

[13] Bonaca M.P., Olenchock B.A., Salem J.E., Wiviott S.D., Ederhy S., Cohen A., Stewart G.C., Choueiri T.K., Di Carli M., Allenbach Y. (2019). Myocarditis in the Setting of Cancer Therapeutics: Proposed Case Definitions for Emerging Clinical Syndromes in Cardio-Oncology. Circulation.

[14] Herrmann J. (2020). Adverse Cardiac Effects of Cancer Therapies: Cardiotoxicity and Arrhythmia. Nat. Rev. Cardiol..

[15] Fradley M.G., Beckie T.M., Brown S.A., Cheng R.K., Dent S.F., Nohria A., Patton K.K., Singh J.P., Olshansky B. (2021). Recognition, Prevention, and Management of Arrhythmias and Autonomic Disorders in Cardio-Oncology: A Scientific Statement from the American Heart Association. Circulation.

[16] Touyz R.M., Herrmann J. (2018). Cardiotoxicity with Vascular Endothelial Growth Factor Inhibitor Therapy. NPJ Precis. Oncol..

[17] Li W., Cornell R.F., Lenihan D., Slosky D., Jagasia M., Piazza G., Moslehi J. (2016). Cardiovascular Complications of Novel Multiple Myeloma Treatments. Circulation.

[18] More L.A., Lane S., Asnani A. (2021). 5-FU Cardiotoxicity: Vasospasm, Myocarditis, and Sudden Death. Curr. Cardiol. Rep..

[19] Ell P., Martin J.M., Cehic D.A., Ngo D.T.M., Sverdlov A.L. (2021). Cardiotoxicity of Radiation Therapy: Mechanisms, Management, and Mitigation. Curr. Treat. Options Oncol..

[20] Giza D.E., Boccalandro F., Lopez-Mattei J., Iliescu G., Karimzad K., Kim P., Iliescu C. (2017). Ischemic Heart Disease: Special Considerations in Cardio-Oncology. Curr. Treat. Options Cardiovasc. Med..

[21] Dobson R., Ghosh A.K., Ky B., Marwick T., Stout M., Harkness A., Steeds R., Robinson S., Oxborough D., Adlam D. (2021). BSE and BCOS Guideline for Transthoracic Echocardiographic Assessment of Adult Cancer Patients Receiving Anthracyclines and/or Trastuzumab. JACC CardioOncol..

[22] Sartorio A., Cristin L., Pont C.D., Farzaneh-Far A., Romano S. (2025). Global Longitudinal Strain as an Early Marker of Cardiac Damage after Cardiotoxic Medications, a State of the Art Review. Prog. Cardiovasc. Dis..

[23] Thavendiranathan P., Poulin F., Lim K.D., Plana J.C., Woo A., Marwick T.H. (2014). Use of Myocardial Strain Imaging by Echocardiography for the Early Detection of Cardiotoxicity in Patients during and after Cancer Chemotherapy: A Systematic Review. J. Am. Coll. Cardiol..

[24] Oikonomou E.K., Kokkinidis D.G., Kampaktsis P.N., Amir E.A., Marwick T.H., Gupta D., Thavendiranathan P. (2019). Assessment of Prognostic Value of Left Ventricular Global Longitudinal Strain for Early Prediction of Chemotherapy-Induced Cardiotoxicity: A Systematic Review and Meta-Analysis. JAMA Cardiol..

[25] Liu J.E., Barac A., Thavendiranathan P., Scherrer-Crosbie M. (2020). Strain Imaging in Cardio-Oncology. JACC CardioOncol..

[26] Nazir M.S., Okafor J., Murphy T., Andres M.S., Ramalingham S., Rosen S.D., Chiribiri A., Plein S., Prasad S., Mohiaddin R. (2024). Echocardiography versus Cardiac MRI for Measurement of Left Ventricular Ejection Fraction in Individuals with Cancer and Suspected Cardiotoxicity. Radiol. Cardiothorac. Imaging.

[27] Lorenzo-Esteller L., Ramos-Polo R., Pons Riverola A., Morillas H., Berdejo J., Pernas S., Pomares H., Asiain L., Garay A., Martinez Perez E. (2024). Pericardial Disease in Patients with Cancer: Clinical Insights on Diagnosis and Treatment. Cancers.

[28] Yalta K., Yilmaztepe M., Zorkun C. (2018). Left Ventricular Dysfunction in the Setting of Takotsubo Cardiomyopathy: A Review of Clinical Patterns and Practical Implications. Card. Fail. Rev..

[29] Addison D., Neilan T.G., Barac A., Scherrer-Crosbie M., Okwuosa T.M., Plana J.C., Reding K.W., Taqueti V.R., Yang E.H., Zaha V.G. (2023). Cardiovascular Imaging in Contemporary Cardio-Oncology: A Scientific Statement from the American Heart Association. Circulation.

[30] Thavendiranathan P., Zhang L., Zafar A., Drobni Z.D., Mahmood S.S., Cabral M., Awadalla M., Nohria A., Zlotoff D.A., Thuny F. (2021). Myocardial T1 and T2 Mapping by Magnetic Resonance in Patients with Immune Checkpoint Inhibitor-Associated Myocarditis. J. Am. Coll. Cardiol..

[31] Ferreira V.M., Schulz-Menger J., Holmvang G., Kramer C.M., Carbone I., Sechtem U., Kindermann I., Gutberlet M., Cooper L.T., Liu P. (2018). Cardiovascular Magnetic Resonance in Nonischemic Myocardial Inflammation. J. Am. Coll. Cardiol..

[32] Wang T.K.M., Jellis C.L., Cremer P.C., Bolen M.A., Flamm S.D., Klein A.L. (2022). Cardiac Magnetic Resonance Imaging Techniques and Applications for Pericardial Diseases. Circ. Cardiovasc. Imaging.

[33] Lopez-Mattei J.C., Yang E.H., Ferencik M., Baldassarre L.A., Dent S., Budoff M.J. (2021). Cardiac Computed Tomography in Cardio-Oncology. JACC CardioOncol..

[34] Erthal F., Premaratne M., Yam Y., Chen L., Lamba J., Keenan M., Haddad T., Pharasi K., Anand S., Beanlands R.S. (2018). Appropriate Use Criteria for Cardiac Computed Tomography: Does Computed Tomography Have Incremental Value in All Appropriate Use Criteria Categories?. J. Thorac. Imaging.

[35] Milazzo V., Cosentino N., Campodonico J., Lucci C., Cardinale D., Cipolla C.M., Marenzi G. (2020). Characteristics, Management, and Outcomes of Acute Coronary Syndrome Patients with Cancer. J. Clin. Med..

[36] Driessen R.S., Danad I., Stuijfzand W.J., Raijmakers P.G., Schumacher S.P., Van Diemen P.A., Leipsic J.A., Knuuti J., Underwood S.R., Van De Ven P.M. (2019). Comparison of Coronary Computed Tomography Angiography, Fractional Flow Reserve, and Perfusion Imaging for Ischemia Diagnosis. J. Am. Coll. Cardiol..

[37] Tyebally S., Chen D., Bhattacharyya S., Mughrabi A., Hussain Z., Manisty C., Westwood M., Ghosh A.K., Guha A. (2020). Cardiac Tumors. JACC CardioOncol..

[38] Angeli F., Bodega F., Bergamaschi L., Armillotta M., Amicone S., Canton L., Fedele D., Suma N., Cavallo D., Foà A. (2024). Multimodality Imaging in the Diagnostic Work-Up of Patients with Cardiac Masses. JACC CardioOncol..

[39] Zhang K.W., Finkelman B.S., Gulati G., Narayan H.K., Upshaw J., Narayan V., Plappert T., Englefield V., Smith A.M., Zhang C. (2018). Abnormalities in 3-Dimensional Left Ventricular Mechanics with Anthracycline Chemotherapy Are Associated with Systolic and Diastolic Dysfunction. JACC Cardiovasc. Imaging.

[40] Camilli M., Ferdinandy P., Salvatorelli E., Menna P., Minotti G. (2024). Anthracyclines, Diastolic Dysfunction and the Road to Heart Failure in Cancer Survivors: An Untold Story. Prog. Cardiovasc. Dis..

[41] Sawyer D.B., Zuppinger C., Miller T.A., Eppenberger H.M., Suter T.M. (2002). Modulation of Anthracycline-Induced Myofibrillar Disarray in Rat Ventricular Myocytes by Neuregulin-1beta and Anti-erbB2: Potential Mechanism for Trastuzumab-Induced Cardiotoxicity. Circulation.

[42] Georgiopoulos G., Makris N., Laina A., Theodorakakou F., Briasoulis A., Trougakos I.P., Dimopoulos M.A., Kastritis E., Stamatelopoulos K. (2023). Cardiovascular Toxicity of Proteasome Inhibitors: Underlying Mechanisms and Management Strategies: JACC: CardioOncology State-of-the-Art Review. JACC CardioOncol..

[43] Senechal I., Andres M.S., Tong J., Ramalingam S., Nazir M.S., Rosen S.D., Young K., Idaikkadar P., Larkin J., Lyon A.R. (2024). Risk Stratification, Screening and Treatment of BRAF/MEK Inhibitors-Associated Cardiotoxicity. Curr. Oncol. Rep..

[44] Mincu R.I., Mahabadi A.A., Michel L., Mrotzek S.M., Schadendorf D., Rassaf T., Totzeck M. (2019). Cardiovascular Adverse Events Associated with BRAF and MEK Inhibitors: A Systematic Review and Meta-Analysis. JAMA Netw. Open.

[45] Chu T.F., Rupnick M.A., Kerkela R., Dallabrida S.M., Zurakowski D., Nguyen L., Woulfe K., Pravda E., Cassiola F., Desai J. (2007). Cardiotoxicity Associated with Tyrosine Kinase Inhibitor Sunitinib. Lancet.

[46] Santoni M., Guerra F., Conti A., Lucarelli A., Rinaldi S., Belvederesi L., Capucci A., Berardi R. (2017). Incidence and Risk of Cardiotoxicity in Cancer Patients Treated with Targeted Therapies. Cancer Treat Rev.

[47] Webster R.M. (2014). The Immune Checkpoint Inhibitors: Where Are We Now?. Nat Rev Drug Discov.

[48] Sterner R.C., Sterner R.M. (2021). CAR-T Cell Therapy: Current Limitations and Potential Strategies. Blood Cancer J..

[49] Lefebvre B., Kang Y., Smith A.M., Frey N.V., Carver J.R., Scherrer-Crosbie M. (2020). Cardiovascular Effects of CAR T Cell Therapy: A Retrospective Study. JACC CardioOncol..

[50] Bayraktar U.D., Bashir Q., Qazilbash M., Champlin R.E., Ciurea S.O. (2013). Fifty Years of Melphalan Use in Hematopoietic Stem Cell Transplantation. Biol. Blood Marrow Transplant..

[51] Feliz V., Saiyad S., Ramarao S.M., Khan H., Leonelli F., Guglin M. (2011). Melphalan-Induced Supraventricular Tachycardia: Incidence and Risk Factors. Clin. Cardiol..

[52] Dhesi S., Chu M.P., Blevins G., Paterson I., Larratt L., Oudit G.Y., Kim D.H. (2013). Cyclophosphamide-Induced Cardiomyopathy: A Case Report, Review, and Recommendations for Management. J. Investig. Med. High Impact Case Rep..

[53] Kamphuis J.A.M., Linschoten M., Cramer M.J., Gort E.H., van Rhenen A., Asselbergs F.W., Doevendans P.A., Teske A.J. (2019). Cancer Therapy-Related Cardiac Dysfunction of Nonanthracycline Chemotherapeutics: What Is the Evidence?. JACC CardioOncol..

[54] Gongora C.A. (2024). Are Sodium-Glucose Cotransporter-2 Inhibitors the Cherry on Top of Cardio-Oncology Care?. Cardiovasc. Drugs Ther..

[55] Gongora C.A., Drobni Z.D., Quinaglia Araujo Costa Silva T., Zafar A., Gong J., Zlotoff D.A., Gilman H.K., Hartmann S.E., Sama S., Nikolaidou S. (2022). Sodium-Glucose Co-Transporter-2 Inhibitors and Cardiac Outcomes Among Patients Treated with Anthracyclines. J. Am. Coll. Cardiol. Heart Fail..

[56] Maamari D.J., Biddinger K.J., Jurgens S.J., Rämö J.T., Zheng A., Hayes D., Gongora C.A., Choi S.H., Arany Z., Thavendiranathan P. (2025). Polygenic Susceptibility to Dilated Cardiomyopathy Underlies Peripartum, Alcohol-Induced, and Cancer Therapy-Related Cardiomyopathies. medRxiv.

